# Integrative Analysis of Metabolome and Microbiome in Patients with Progressive Alcohol-Associated Liver Disease

**DOI:** 10.3390/metabo11110766

**Published:** 2021-11-10

**Authors:** Bei Gao, Yixin Zhu, Nan Gao, Weishou Shen, Peter Stärkel, Bernd Schnabl

**Affiliations:** 1 School of Marine Sciences, Nanjing University of Information Science and Technology, Nanjing 210044, China; wintergb@hotmail.com; 2 Department of Medicine, University of California San Diego, La Jolla, CA 92093, USA; y3zhu@ucsd.edu; 3 School of Biological and Pharmaceutical Engineering, Nanjing Tech University, Nanjing 211816, China; ngao@njtech.edu.cn; 4 School of Environmental Science and Engineering, Nanjing University of Information Science and Technology, Nanjing 210044, China; wsshen@nuist.edu.cn; 5 Jiangsu Key Laboratory of Atmospheric Environment Monitoring and Pollution Control, Collaborative Inovation Center of Atmospheric Environment and Equipment Technology, Nanjing 210044, China; 6 Laboratory of Hepato-Gastroenterology, Institute of Experimental and Clinical Research, Université Catholique de Louvain, 1200 Brussels, Belgium; 7 Department of Hepato-Gastroenterology, St. Luc University Hospital, Université Catholique de Louvain, 1200 Brussels, Belgium; 8 Department of Medicine, VA San Diego Healthcare System, San Diego, CA 92161, USA

**Keywords:** gut microbiota, random forest, metabolome, multi-omics

## Abstract

Alcohol-associated liver disease is one of the most prevalent diseases around the world, with 10–20% of patients developing progressive liver disease. To identify the complex and correlated nature of metabolic and microbial data types in progressive liver disease, we performed an integrated analysis of the fecal and serum metabolomes with the gut microbiome in a cohort of 38 subjects, including 15 patients with progressive liver disease, 16 patients with non-progressive liver disease, and 7 control subjects. We found that although patients were generally clustered in three groups according to disease status, metabolites showed better separation than microbial species. Furthermore, eight serum metabolites were correlated with two microbial species, among which seven metabolites were decreased in patients with progressive liver disease. Five fecal metabolites were correlated with three microbial species, among which four metabolites were decreased in patients with progressive liver disease. When predicting progressive liver disease from non-progressive liver disease using correlated metabolic and microbial signatures with the random forest model, correlated serum metabolites and microbial species showed great predictive power, with the area under the receiver operating characteristic curve achieving 0.91. The multi-omics signatures identified in this study are helpful for the early identification of patients with progressive alcohol-associated liver disease, which is a key step for therapeutic intervention.

## 1. Introduction

Alcohol-associated liver disease (ALD) is one of the most prevalent diseases around the world and is a global health burden [1,2,3]. Chronic alcohol consumption is the main contributor to ALD development [1,2]. Other factors, such as gender, ethnicity, metabolic syndrome, concomitant liver diseases, genetic factors, and smoking history, can also affect the progression of ALD [1,2]. ALD includes a wide spectrum of liver manifestations, from simple steatosis developing in the majority of patients with alcohol use disorder, to progressive steatohepatitis, fibrosis, and cirrhosis developing in 10–20% of patients. Early identification of progressive liver disease is a key step for effective intervention.

Chronic alcohol consumption or binge drinking significantly alters the gut microbial profile, resulting in increased bacterial translocation and intestinal barrier dysfunction, which promotes ALD development [4,5,6,7,8,9,10,11,12]. While abstinence is the most important factor for ALD treatment, the use of probiotics to alter the gut microbiota might be effective in the prevention and treatment of ALD [13,14,15,16,17]. Metabolomics studies are conducted to determine host or bacteria-associated metabolites as biomarkers of ALD [8,18]. For instance, changes in the gut microbiota affect bile acid metabolism and induce inflammation and liver injury in ALD patients [19,20,21,22,23,24].

While technological advances have led to multiple omics datasets, large-scale multi-omics integration is still a challenging task. While there are many software tools available to analyze metabolomics or microbial datasets, most of them are only accountable for analyzing single omics datasets. However, investigating a single omics dataset is oftentimes insufficient to fully understand the biological significance of gut microbes and metabolites. Here, we used mixOmics to perform an integrative and comprehensive analysis of the metabolome and gut microbiome in ALD patients, which incorporates a wide range of methods to support multi-omics analysis [25]. We investigated the complex and correlated nature of metagenomics and metabolomics data, which are used as multi-omics signatures to predict progressive liver disease in ALD patients.

## 2. Results

### 2.1. Patient Characteristics

We used mixOmics to perform an integrative analysis of the metabolome and microbial species in 38 subjects, including 15 patients with progressive liver disease, 16 patients with non-progressive liver disease, and 7 control subjects. Subject characteristics are shown in Table 1. As expected, patients with progressive liver disease showed higher levels of alanine aminotransferase (ALT), aspartate aminotransferase (AST), total bilirubin, gamma-glutamyl-transferase (GGT), controlled attenuation parameter (CAP), and CK18-M65.

### 2.2. Microbial Species, Fecal and Serum Metabolites in ALD Patients

**Figure 1:**
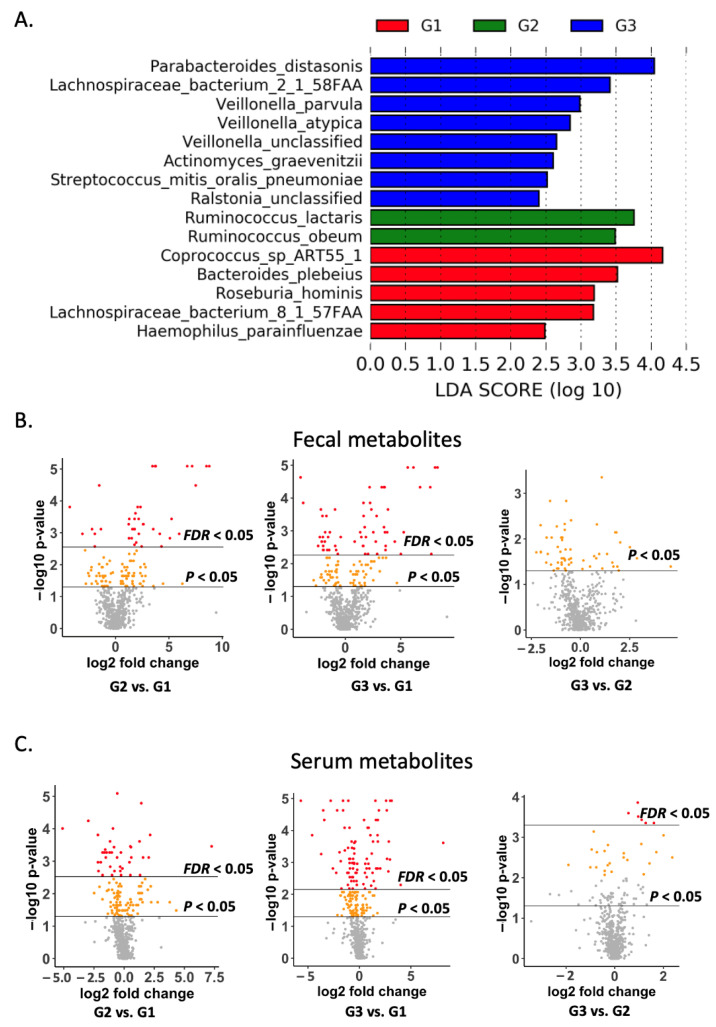
Microbial species, fecal and serum metabolites in ALD patients: (**A**) significant microbial species; (**B**) volcano plot of fecal metabolites; (**C**) volcano plot of serum metabolites. G1: non-alcoholic control subjects; G2: patients with alcohol-associated non-progressive liver disease; G3: patients with alcohol-associated progressive liver disease. FDR: false discovery rate.

A total of 280 microbial species were detected in ALD patients. As shown in Figure 1A, five, two, and eight microbial species were enriched in control subjects, patients with alcohol-associated non-progressive liver disease, and patients with progressive liver disease, respectively, as revealed by Linear discriminant analysis Effect Size (LEfSe).

A total of 590 metabolites were annotated in the fecal samples. When comparing patients with non-progressive liver disease to control subjects, 130 fecal metabolites showed a raw *p*-value of less than 0.05, among which 39 metabolites showed a false discovery rate (FDR) of less than 0.05 (Figure 1B, left). When comparing patients with progressive liver disease to control subjects, 130 metabolites showed a raw *p*-value of less than 0.05, among which 60 metabolites showed an FDR of less than 0.05 (Figure 1B, middle). When comparing patients with progressive liver disease to patients with non-progressive liver disease, 58 metabolites showed a raw *p*-value of less than 0.05, among which none showed an FDR of less than 0.05 (Figure 1B, right).

A total of 590 metabolites were also annotated in the serum samples. When comparing patients with non-progressive liver disease to control subjects, 123 serum metabolites showed a raw *p*-value of less than 0.05, among which 39 metabolites showed an FDR less than 0.05 (Figure 1C, left). When comparing patients with progressive liver disease to control subjects, 170 metabolites showed a raw *p*-value of less than 0.05, among which 89 metabolites showed an FDR of less than 0.05 (Figure 1C, middle). When comparing patients with progressive liver disease to patients with non-progressive liver disease, 78 metabolites showed a raw *p*-value of less than 0.05, among which six metabolites showed an FDR of less than 0.05 (Figure 1C, right).

### 2.3. Correlation between Fecal Metabolites and Microbial Species

**Figure 2:**
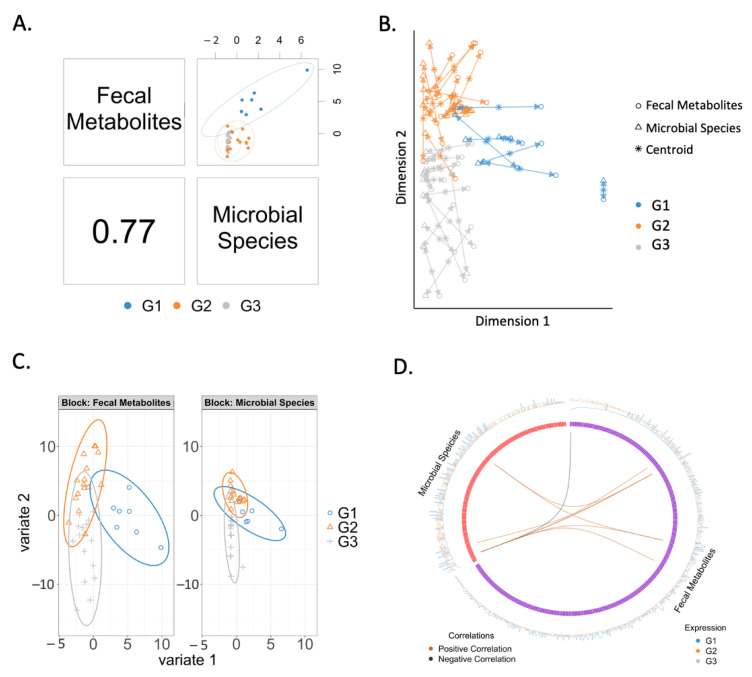
Correlated fecal metabolites and microbial species: (**A**) The overall correlation between fecal metabolites and microbial species is 0.77. (**B**) Similarities (points are clustered) and dissimilarities between samples and groups. Samples are represented as points placed according to their projection in the smaller subspace spanned by microbial species and serum metabolites. (**C**) Agreement between microbial species and fecal metabolites. Each arrow corresponds to one sample. The start of the arrow indicates the location of the sample in the microbial species plot, and the tip is the location of the sample in the fecal metabolites plot. Short arrows indicate if both data sets strongly agree, and long arrows indicate a disagreement between two data sets. (**D**) Correlations between variables of serum metabolites and microbial species. Cut-off is set to 0.6. A black line indicates a negative correlation; an orange line indicates a positive correlation. G1: non-alcoholic control subjects; G2: patients with alcohol-associated non-progressive liver disease; G3: patients with alcohol-associated progressive liver disease.

The overall correlation between fecal metabolites and microbial species was 0.77 (Figure 2A). The agreement between microbial species and fecal metabolites is shown in Figure 2B. When analyzing fecal metabolites and microbial species separately, we found that both microbial species and fecal metabolites were generally clustered in three groups according to the disease status, with fecal metabolites showing better separation than microbial species (Figure 2C). The correlation between fecal metabolites and microbial species is shown in the circos plot (Figure 2D, Table 2). *Coprococcus* sp. ART55/1 was negatively correlated with the fecal level of phenylalanine, and positively correlated with the fecal level of 3-methylglutarylcarnitine, carbamazepine, linolenic acid, and cystine. Meanwhile, *Acidaminococcus fermentans* was positively correlated with linolenic acid and cystine levels in feces. *Lachnospiraceae bacterium* 8_1_57FAA was positively correlated with the fecal level of linolenic acid.

### 2.4. Correlation between Serum Metabolites and Microbial Species

**Figure 3:**
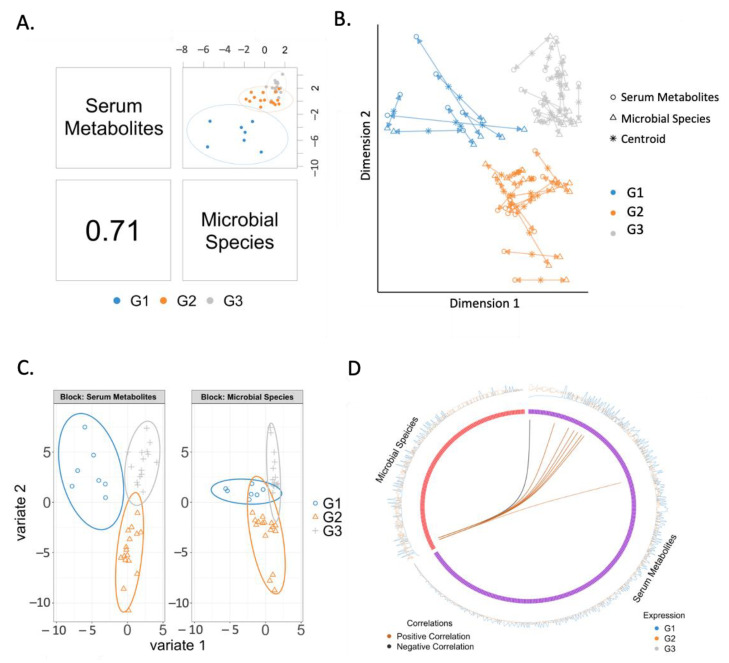
Correlated serum metabolites and microbial species. (**A**) The overall correlation between serum metabolites and microbial species is 0.71. (**B**) Similarities (points are clustered) and dissimilarities between samples and groups. Samples are represented as points placed according to their projection in the smaller subspace spanned by microbial species and serum metabolites. (**C**) Agreement between microbial species and serum metabolites. Each arrow corresponds to one sample. The start of the arrow indicates the location of the sample in the microbial species plot, and the tip is the location of the sample in the serum metabolites plot. Short arrows indicate if both data sets strongly agree, and long arrows indicate a disagreement between two data sets. (**D**) Correlations between variables of serum metabolites and microbial species. Cut-off is set to 0.6. A black line indicates a negative correlation; an orange line indicates a positive correlation. G1: non-alcoholic control subjects; G2: patients with alcohol-associated non-progressive liver disease; G3: patients with alcohol-associated progressive liver disease.

The overall correlation between serum metabolites and microbial species was 0.71 (Figure 3A). In particular microbial species and serum metabolites from patients with progressive liver disease showed better agreement than the two other groups (Figure 3B). When analyzing serum metabolites and microbial species separately, we found that both microbial species and serum metabolites were generally clustered by disease status, with serum metabolites showing better separation than microbial species (Figure 3C). As shown in the circos plot, correlations were found between eight serum metabolites and two microbial species (Figure 3D, Table 3). *Odoribacter splanchnicus* and *Coprococcus* sp. ART55-1 were negatively correlated with the serum level of glutamic acid, but positively correlated with 2-O-methylcytidine, 3-hydroxyanthranilic acid, glutamine, guanosine, inosine, and kynurenic acid. In addition, *Coprococcus* sp. ART55-1 was positively correlated with the serum level of butyrylcarnitine.

### 2.5. Changes in Correlated Metabolites and Gut Microbes in Three Groups

**Figure 4:**
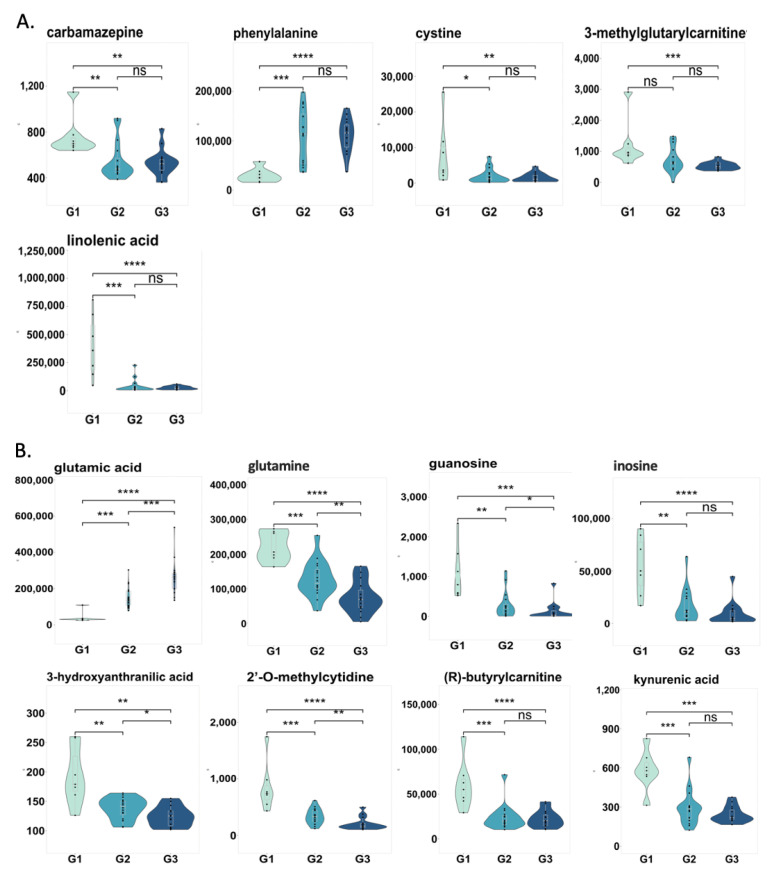
Changes in correlated metabolites and microbial species in three groups. (**A**) Fecal metabolites. (**B**) Serum metabolites. G1: non-alcoholic control subjects; G2: patients with alcohol-associated non-progressive liver disease; G3: patients with alcohol-associated progressive liver disease. ns: *p* > 0.05; *: *p* < 0.05; **: *p* < 0.01; ***: *p* < 0.001; ****: *p* < 0.0001.

Next, we examined the levels of five fecal metabolites in three groups correlated with microbial species. Out of five fecal metabolites, three were significantly decreased in patients with both non-progressive liver disease and progressive liver disease compared to control subjects (Figure 4A). Meanwhile, the level of 3-methylglutarylcarnitine was significantly decreased in patients with progressive liver disease compared to control subjects. In contrast, phenylalanine was increased in patients with both non-progressive liver disease and progressive liver disease compared to controls. We further examined the level of eight serum metabolites, which showed correlations with microbial species in the three groups, as shown in Figure 4B. Out of eight serum metabolites, seven were significantly decreased in patients with both non-progressive liver disease and progressive liver disease compared to controls. Among these seven metabolites, the levels of four were significantly decreased in patients with progressive liver disease compared to patients with non-progressive liver disease, including glutamine, 3-hydroxyanthanilic acid, 2′-O-methylcytidine, and guanosine. In contrast to glutamine, glutamic acid was increased in patients with both non-progressive liver disease and progressive liver disease. Notably, glutamic acid was significantly increased in patients with progressive liver disease compared to patients with non-progressive liver disease. Next, we checked the microbial pathways that are associated with these metabolites in three groups (Table S1). However, no significant difference was found in these pathways.

Among four microbial species correlated with serum or fecal metabolites, *Coprococcus* sp. ART55-1 and *Lachnospiraceae bacterium* 8_1_57FAA were enriched in control subjects; meanwhile, the levels of *Acidaminococcus fermentans* and *Odoribacter splanchnicus* were not significantly different among the three groups (Figure 1A).

### 2.6. Prediction of Progressive Liver Disease Using Correlated Metabolites and Microbial Species

**Figure 5:**
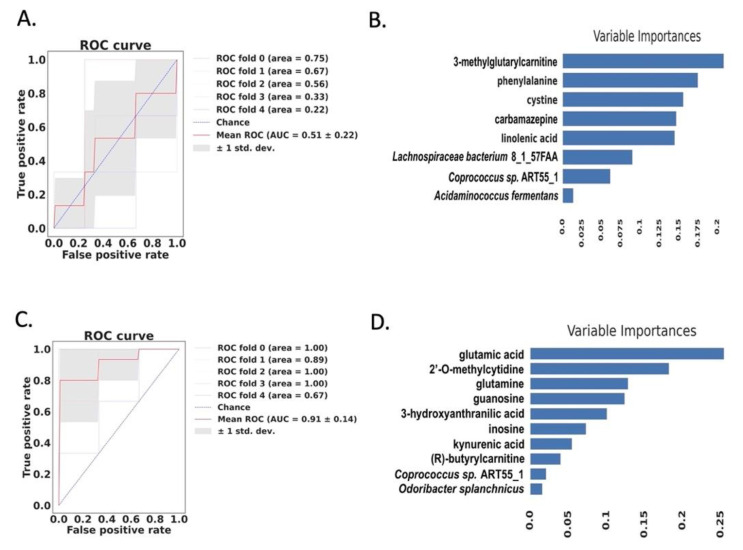
Prediction of progressive liver disease using random forest model. (**A**) Prediction of progressive alcohol-associated liver disease using correlated fecal metabolites and microbial species. (**B**) Variable importance of correlated fecal metabolites and microbial species. (**C**) Prediction of progressive liver disease using correlated serum metabolites and microbial species. (**D**) Variable importance of serum metabolites and microbial species.

We built a random forest model to differentiate patients with progressive liver disease from patients with non-progressive liver disease. When using correlated fecal metabolites and microbial species, the area under receiver operating characteristic (AUROC) curve was only 0.51 (Figure 5A). The importance of correlated variables is shown in Figure 5B. Correlated serum metabolites and microbial species showed strong predictive power, with an AUROC curve of 0.91 when predicting progressive liver disease (Figure 5C), which is better than that of correlated fecal metabolites and microbial species. The importance of ten variables is shown in Figure 5D. The serum level of glutamic acid was selected as the most important variable for the prediction. Overall, serum metabolites perform better than two microbial species when predicting progressive liver disease.

## 3. Discussion

Host and microbiota-derived metabolites in the gut lumen translocate to the liver through the portal vein. Gut barrier dysfunction was found in patients with ALD, which facilitates the translocation of host and microbiota-derived metabolites to the portal vein and systemic circulation. In this study, we performed integrated analysis of metabolomics and metagenomics to identify correlated multi-omics features in patients with ALD. With the implementation of DIABLO, the process of revealing correlated microbes with fecal or serum metabolites is facilitated in our study. This is further used to predict the progression of ALD simply based upon selected microbes and metabolites using the random forest model.

As one of the most popular ensemble techniques of classification, random forest has emerged as a potential tool for clinical decision making, which typically uses decision trees as base classifiers and “combines” them in an iterative fashion. At each iteration, a new decision tree is trained with respect to the misclassification error obtained from the last iteration, and the iterative procedure stops when the reduction in the misclassification error is below a pre-assigned value [26].

Glutamic acid was selected as the most important variable to predict the progressive liver disease in our random forest model, which was increased in the serum of patients with both non-progressive liver disease patients and progressive liver disease, compared to the serum of the control group. In patients with alcohol-associated liver cirrhosis, plasma and ascitic fluid show an elevated concentration of glutamic acid compared to normal controls [27]. An increase in glutamic acid concentration in serum or plasma might contribute to the severity of non-alcoholic fatty liver disease (NAFLD), non-alcoholic steatohepatitis (NASH), and liver fibrosis [28,29]. In contrast to the increase in glutamic acid, the serum levels of glutamine were decreased in patients with non-progressive and progressive liver disease as compared to non-alcoholic controls. Chronic alcohol consumption has been reported to down-regulate the biosynthesis of glutamine [30]. A previous study found lower plasma glutamine levels in patients with alcohol-associated liver disease as compared to non-alcoholic liver disease controls [31]. A glutamine-supplemented diet prevents ethanol-induced liver injury in a mouse model [32,33].

An intermediate product of tryptophan degradation, 3-hydroxyanthranilic acid, was decreased in the serum of both non-progressive liver disease and progressive liver disease patients in our study. The tryptophan metabolism rate is low due to insufficient vitamins, which potentially results in low 3-hydroxyanthranilic acid production in the serum of patients with severe liver disease [34]. Similar to 3-hydroxyanthranilic acid, kynurenic acid is also a key intermediate product of tryptophan degradation [35]. Here, serum kynurenic acid showed the same decrease in both patient groups.

Linolenic acid belongs to the omega-3 (n-3) polyunsaturated fatty acids (PUFAs) family. In our study, compared to the control group, a decreased linolenic acid level was found in the feces of both non-progressive liver disease patients and progressive liver disease patients. Previous findings have reported a negative correlation between the concentration of linolenic acid and the severity of cirrhosis in patients [36]. Consuming a linolenic acid-rich diet may act as an effective way of preventing ALD [37,38].

Among the microbial species that showed a correlation with fecal or serum metabolites in our study, *Coprococcus* sp. ART55-1 and *Lachnospiraceae bacterium* 8_1_57FAA were significantly enriched in control subjects. *Coprococcus* was inversely associated with steatosis in a large-scale study of 1355 adults [39]. *Lachnospiraceae bacterium* is known as one of the most dominant bacteria taxa present in the human gut microbiota [40]. A previous finding reported a decreased abundance level in Lachnospiraceae in the feces of alcoholic hepatitis patients compared to healthy controls and heavy-drinking subjects [41]. In a study about chronic hepatitis B virus (HBV), the abundance of Lachnospiraceae was significantly reduced in HBV patients who consumed alcohol as compared to HBV patients who did not [42]. In addition, Lachnospiraceae has also been reported to be correlated with lung diseases and HIV [43,44].

Our study design is cross-sectional in nature, which aims at investigating the metabolites and microbial species that potentially contribute to the development of progressive alcohol-associated liver disease in ALD patients. Unlike longitudinal studies, our data and patient information were gathered at a single timepoint. With such data structure, a significant association between metabolites and microbial species can be easily identified in a short amount of time [45]. However, the causal relationship remains unknown and requires further investigation. Due to the sample availability, the sample size of this study is relatively small. The findings from this study need to be validated in a larger patient cohort.

In conclusion, we performed an integrated analysis of metabolomics and metagenomics in ALD patients, revealing multiple correlated metabolites and gut microbes. Correlated serum metabolites and microbes show great potential for the prediction of progressive liver disease. The key metabolites and gut microbes identified in our findings could be used in clinical practice to predict the progression of liver disease, which is helpful for patient stratification and possibly for the development of treatment strategies. The findings in the present study provide a solid foundation for future studies to investigate the mechanisms behind such correlations and their contribution to the progression of ethanol-induced liver disease in preclinical models. The research approach presented in this study could serve as a starting point for further longitudinal studies and the evaluation of therapeutic options for ALD.

## 4. Materials and Methods

### 4.1. Patients

A total of 31 patients who met the Diagnostic and Statistical Manual of Mental Disorders, Fourth Edition criteria, were recruited for the study. The patients consumed alcohol (>60 g/day) for more than one year and were actively drinking until the day of admission for detoxification. Based on clinical parameters, patients were split into two groups. Sixteen patients were defined as having non-progressive liver disease (minimal liver injury and simple steatosis), with normal ALT/AST (<40 U/L), liver stiffness < 7.6 kPa, and CAP < 250 dB/m (minimal liver injury). A CAP greater than 250 dB/m was allowed if all other criteria were normal. Fifteen patients were defined as having progressive liver disease (steatohepatitis and steatofibrosis), with increased ALT/AST (>40 U/L) and one or more of the following parameters: liver stiffness > 7.6 kPa (significant fibrosis), and/or CAP > 250 dB/m. CK18-M65 blood levels with a 400 U/L cut-off were used to support the classification of non-progressive and progressive ALD [46]. Seven non-alcoholic controls were recruited who consumed less than 20 g of alcohol per day. Controls were matched for gender, age, and BMI. During the two months preceding enrollment, patients and control subjects did not take immunosuppressive medication or antibiotics. The study protocol was approved by the human research and ethical committee of the Université Catholique de Louvain, Brussels, Belgium (B403201422657). Written informed consent was obtained from all patients and control subjects after the nature and possible consequences of the studies were explained.

### 4.2. Untargeted Metabolomics

The serum and fecal metabolome from 38 subjects were analyzed by gas chromatography–time of flight mass spectrometry (GC–TOF MS) and hydrophilic interaction liquid chromatography (HILIC) with quadrupole orbital ion trap high field mass spectrometry (Q-Exactive HF MS). Sample extraction, data acquisition, and data processing were performed as described in our previous study [47]. Briefly, ChromaTOF version 4.50 and Binbase version 5.0.3 were used for GC-MS data processing [48]. For LC-MS raw data, MS-DIAL [49] and MS-FLO [50] were used for LC-MS data processing. For the HILIC dataset, retention time-m/z libraries and the MS/MS spectra database were used for compound identification, which were uploaded to MassBank of North America.

### 4.3. Shotgun Metagenomics

DNA was extracted from stool samples collected from the same 38 subjects. DNA extraction and library preparation were performed as described previously [51]. Shot-gun metagenomics sequencing was performed on Illumina HiSeq 4000 generating 150 bp paired-end reads. KneadData version 0.7.2 was used for the quality control of raw sequencing data. Metagenomic Phylogenetic Analysis 2 (MetaPhlAn2) version 2.7.7 [52] was used for the profiling the composition of microbial communities. HMP Unified Metabolic Analysis Network 2 (HUMAnN2) version 0.11.1 was used for the profiling of microbial pathways [53]. The MetaCyc database was used for microbial pathway analysis [54].

### 4.4. Integrative Analysis of Microbiota and Metabolomics Data

An integrative metagenomics and metabolomics analysis was performed using mixOmics (version 6.14.1) [25,55], which is able to achieve a similar performance with improved insights in prediction compared to other state-of-the-art models [56]. The design matrix for both fecal metabolites with microbial species (0.15) and serum metabolites with microbial species (0.01) was refined according to the Projection to Latent Structure (PLS) model correlation. The Data Integration Analysis for Biomarker discovery using a Latent component method for Omics studies (DIABLO) model was fitted to our data with a 10-fold cross-validation repeated 10 times and then tuned with the tune.block.splsda() method. The DIABLO framework is designed for multi-omics analysis for sample group discrimination and class prediction to identify novel biomarkers [56]. We used plotDiablo(), plotIndiv(), and plotArrow() with default parameters for data visualization. A circos plot was generated using circosPlot() with a cut-off value of 0.6.

### 4.5. Statistical Analysis

R (version 4.0.2) was used for the statistical analysis. The Kruskal–Wallis test was used to calculate the significance between three groups of metabolomics data, and the Mann–Whitney Wilcoxon test was used to calculate the significance between the two groups. LEfSe was used to determine the microbial species most likely to explain the difference between three groups [57]. The H_2_O platform (https://www.h2o.ai, accessed on 6 September 2021) was used to build the random forest model for predicting progressive liver disease using correlated metabolites and microbial species. The datasets were split into training and test datasets (80:20 stratified splits). The model was tuned by performing stratified 5-fold cross-validation on the training set.

## Figures and Tables

**Table 1:** Subject characteristics.

	Non-Alcoholic Controls	Non-Progressive Alcohol-Associated Liver Disease	Progressive Alcohol-Associated Liver Disease	*p*-Value
Clinical parameter				
Total n	7	16	15	
Age, years, n = 38	52 (37–71)	37 (27–58)	42 (28–59)	0.096
Body Mass Index (BMI), kg/m^2^, n = 38	23 (19–29)	22 (19–31)	24 (18–31)	0.437
Gender (male), n (%), n = 38	6 (86)	11 (69)	13 (87)	0.421
Laboratory parameter				
Albumin (g/dL), n = 27		4.7 (4.2–5.2)	4.8 (3.9–5.2)	0.519
ALT (U/L), n = 31		19.5 (11–37)	77 (37–184)	<0.001
AST (U/L), n = 31		25.5 (15–36)	78 (46–283)	<0.001
Total bilirubin (mg/dL), n = 28		0.3 (0.2–1.1)	0.5 (0.3–0.8)	0.049
GGT (U/L), n = 28		31 (4–213)	121 (11–952)	0.021
Platelet counts (×10^9^/L), n = 27		268 (165–339)	222 (21–434)	0.286
Creatinine (mg/dL), n = 28		0.8 (0.5–0.95)	0.8 (0.6–1.2)	0.433
International normalized ratio, n = 28		1.0 (0.9–1.2)	0.9 (0.8–1.0)	0.128
Fibroscan (kpa), n = 30		4.9 (3.1–6.8)	6.1 (3.9–7.0)	0.262
CAP, (dB/m), n = 31 CAP > 250 dB/m, n (%)		254.5 (148–325) 9 (56)	311 (222–381) 15 (93)	0.001
CK18-M65 (U/L), n = 30		332 (158–616)	592 (316–1576)	<0.001

Note: Values are presented as median and range in parentheses ( ). The number of patients for which the respective data were available is indicated in the first column. In blank cells, patients from the respective group were not counted due to missing numbers. Kruskal–Wallis test was used for three group comparisons. Pairwise comparisons were performed using Tukey and Kramer (Nemenyi) test with Tukey-Dist approximation for independent samples. Mann–Whitney test was used for two group comparisons. Bold font indicates significance (*p*-value < 0.05). ALT, alanine aminotransferase; AST, aspartate aminotransferase; GGT, gamma-glutamyl-transferase; CAP, controlled attenuation parameter.

**Table 2:** Correlation between fecal metabolites and microbial species.

	*Acidaminococcus fermentans*	*Lachnospiraceae bacterium* 8_1_57FAA	*Coprococcus* sp. ART55/1
3-methylglutarylcarnitine	0.5513	0.5446	0.6061
carbamazepine	0.5729	0.5593	0.6227
phenylalanine	−0.5991	−0.5645	−0.6292
linolenic acid	0.6671	0.6246	0.6963
cystine	0.6008	0.5393	0.6021

Note: Calculated based on similarity matrix; cut-off 0.6.

**Table 3:** Correlation between serum metabolites and microbial species.

	*Odoribacter splanchnicus*	*Coprococcus* sp. ART55/1
(R)-butyrylcarnitine	0.5975	0.6111
2-O-methylcytidine	0.6520	0.6606
3-hydroxyanthranilic acid	0.6065	0.6182
glutamine	0.6629	0.6681
guanosine	0.6160	0.6256
inosine	0.6376	0.6481
kynurenic acid	0.6291	0.6404
glutamic acid	−0.6085	−0.6075

Note: Calculated based on similarity matrix; cut-off 0.6.

## Data Availability

The sequencing data can be found in the BioProject database (accession: PRJNA613834, https://www.ncbi.nlm.nih.gov/Traces/study/?acc=PRJNA613834).

## Supplementary Materials

The following are available online at https://www.mdpi.com/article/10.3390/metabo11110766/s1, Table S1: Relative abundance of microbial pathways.
