# Supplementary material for: Integrative Analysis of Metabolome and Microbiome in Patients with Progressive Alcohol-Associated Liver Disease
Source: Metabolites. 2021 Nov 10;11(11):766. doi: 10.3390/metabo11110766 (PMC8621614; doi:10.3390/metabo11110766)
Supplement: Supplementary file 1 [file metabolites-11-00766-s001.zip › metabolites-1442117-supplementary.pdf]

Table S1. Relative abundance of microbial pathways

	Control	Non-progressive Liver Disease	Progressive Liver Disease
PWY-6628: superpathway of L-phenylalanine biosynthesis	4.04E-06	1.47E-05	1.13E-05
PWY-5505: L-glutamate and L-glutamine biosynthesis	1.84E-06	2.32E-06	2.88E-06
PWY-5695: urate biosynthesis/inosine 5'-phosphate degradation	3.25E-04	3.02E-04	2.89E-04
PWY-6123: inosine-5'-phosphate biosynthesis I	1.65E-04	1.83E-04	1.77E-04
PWY-6124: inosine-5'-phosphate biosynthesis II	1.44E-04	1.48E-04	1.15E-04
PWY-6549: L-glutamine biosynthesis III	0.00E+00	7.55E-07	3.88E-06
PWY-7234: inosine-5'-phosphate biosynthesis III	1.08E-04	1.06E-04	9.05E-05
PWY-6125: superpathway of guanosine nucleotides de novo biosynthesis II	1.40E-04	1.26E-04	1.69E-04
PWY-6595: superpathway of guanosine nucleotides degradation (plants)	1.10E-05	2.16E-05	1.83E-05
PWY-6608: guanosine nucleotides degradation III	5.00E-05	7.43E-05	7.79E-05
PWY-7221: guanosine ribonucleotides de novo biosynthesis	6.16E-04	6.15E-04	6.22E-04
PWY-7222: guanosine deoxyribonucleotides de novo biosynthesis II	9.63E-05	8.20E-05	1.16E-04
PWY-6606: guanosine nucleotides degradation II	2.06E-05	2.94E-05	3.02E-05

Note: Each of the HUMAnN2 abundance output was normalized into relative abundance (the counts for each sample sum to 100). Values in the table were represented as the median of each metacyc pathway.
